# Improving Network Representation Learning via Dynamic Random Walk, Self-Attention and Vertex Attributes-Driven Laplacian Space Optimization

**DOI:** 10.3390/e24091213

**Published:** 2022-08-30

**Authors:** Shengxiang Hu, Bofeng Zhang, Hehe Lv, Furong Chang, Chenyang Zhou, Liangrui Wu, Guobing Zou

**Affiliations:** 1 School of Computer Engineering and Science, Shanghai University, Shanghai 200444, China; 2 School of Computer and Information Engineering, Shanghai Polytechnic University, Shanghai 201209, China; 3 School of Computer Science and Technology, Kashi University, Kashi 844008, China; 4 School of Information Engineering, Yangzhou Polytechnic Institute, Yangzhou 225127, China

**Keywords:** network representation learning, dynamic random walk, feature extraction, Laplacian space optimization

## Abstract

Network data analysis is a crucial method for mining complicated object interactions. In recent years, random walk and neural-language-model-based network representation learning (NRL) approaches have been widely used for network data analysis. However, these NRL approaches suffer from the following deficiencies: firstly, because the random walk procedure is based on symmetric node similarity and fixed probability distribution, the sampled vertices’ sequences may lose local community structure information; secondly, because the feature extraction capacity of the shallow neural language model is limited, they can only extract the local structural features of networks; and thirdly, these approaches require specially designed mechanisms for different downstream tasks to integrate vertex attributes of various types. We conducted an in-depth investigation to address the aforementioned issues and propose a novel general NRL framework called dynamic structure and vertex attribute fusion network embedding, which firstly defines an asymmetric similarity and *h*-hop dynamic random walk strategy to guide the random walk process to preserve the network’s local community structure in walked vertex sequences. Next, we train a self-attention-based sequence prediction model on the walked vertex sequences to simultaneously learn the vertices’ local and global structural features. Finally, we introduce an attributes-driven Laplacian space optimization to converge the process of structural feature extraction and attribute feature extraction. The proposed approach is exhaustively evaluated by means of node visualization and classification on multiple benchmark datasets, and achieves superior results compared to baseline approaches.

## 1. Introduction

**Figure 1:**
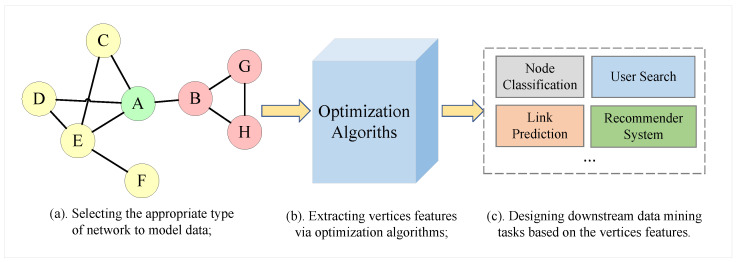
An overview of the three steps of network analysis. First, selection of an appropriate network type to represent the objects and their interrelationships as a starting point. Second, extraction of the features of vertices to retain network structure information, vertex attribute information, and other essential and useful network information [8]. The obtained features are then used for subsequent data-mining tasks.

Network structure can effectively model complex inter-entity relationships, such as social networks between users [1], e-commerce networks between users and products [2], citation networks between publications [3], biological networks [4], etc. These networks have been utilized for a range of data-mining applications, including vertex classification [5], link prediction [6], user search [7] and recommendation systems [2]. Figure 1 depicts the three processes of network data mining.

The second step is the most crucial since the capability of acquired features to characterize the network has a significant effect on the execution of subsequent tasks. Traditional supervised matrix decomposition-based network analysis approaches strongly bind the second and third phases, and employ specialized algorithms for a variety of downstream tasks and network types. However, as information technology advances, networks become more diverse, extensive, and sparse, which makes labeling all vertices, and performing matrix decomposition, impractical and time-consuming. In addition, approach migration is precluded by the great bond between algorithms and downstream tasks. In recent years, unsupervised network representation learning (NRL) approaches [9,10,11,12], which are based on random walk and shallow neural language models, have been extensively researched and have achieved great success. These approaches separate the second and third phases, and focus on extracting representative low-dimensional latent features for vertices. The resulting features can then be directly incorporated into further vector-based data mining algorithms for subsequent tasks. In general, random walks on a network are run to generate vertex sequences that contain information about the network’s structure, and then a neural language model is trained [13] by modeling the co-occurrence of vertices pairs on the sequences to extract structural features for the vertices. Despite the fact that these methods have demonstrated their efficacy, they suffer from the three flaws listed below: First, because the random walk is a Markov process based on a static probability distribution without considering previous walk history, the sampled vertex sequences are prone to losing vertex neighborhood structure information; second, because the feature extraction capability of the shallow language model is limited, only local structural features can be learned, while global structural features are ignored; and third, in order to incorporate the vertex attribute information into network representation, a specific attribute fusion algorithm has to be designed for different downstream tasks and attribute types, which raises the design complexity of the approach.

To address the aforementioned issues, we propose a general NRL approach called dynamic structure and vertex attributes fusion network embedding (dSAFNE). First, based on a newly defined asymmetric second-order vertex approximity, we design an *h*-hop weighted dynamic random walk strategy, which incorporates a series of previously walked vertices to dynamically calculate the sampling probability of each vertex and assign a higher walk probability to the more similar ones. The sampled vertex sequences are then fed into a self-attention-based sequence prediction model. Vertex representations, which preserve both local and global structural characteristics, can be learned through a fake task that predicts the next walked vertex based on the previous vertex sequence. Moreover, in order for our model to effectively extract vertex attribute features of various types, we introduce a vertex attributes-driven Laplacian space optimization. It first leverages the state-of-the-art (SOTA) learning models for different types of data (e.g., residual neural network (ResNet) [14] for images, bidirectional encoder representations from Transformer (BERT) [15] for text, etc.) to extract vertex attribute features. Then, it uses the pairwise feature similarities of the vertices to optimize the vertex representations so that vertices with a higher attribute similarity are represented more closely in the feature space. Finally, we validate the effectiveness of the dSAFNE framework on various datasets through different downstream tasks. This paper’s major contributions are outlined below.

- To address the issue of too much randomness in random walk that is based on a static probability distribution Markov process, we first design an asymmetric second-order proximity that reflects vertex similarity while retaining role information, and then propose an *h*-hop weighted dynamic random walk strategy based on this similarity. It incorporates a series of historical vertices to dynamically calculate the sampling probability of each vertex, and allocates higher walk probabilities to vertices with high similarities, leading to fully capturing the vertex neighborhood structure information.
- To address the issue that the shallow language model adopted by the majority of unsupervised NRL approaches can only extract short-range local structural features of vertices, we model learning network structural features from vertex sequences as a sequence prediction problem and develop a sequence prediction model based on the self-attention mechanism with reference to the structure of the Transformer [16] encoder, which can handle both short-range and long-range dependence problems in order to simultaneously learn the multi-granularity structural features of vertices.
- To address the issue of underutilization of vertex attribute information, we propose a vertex attribute-driven Laplacian optimization that constrains the NRL training process by vertex attribute similarity in order to obtain distributed representations of vertices containing vertex attribute features.
- To evaluate the proposed dSAFNE model, we conducted a series of experiments on vertex classification and visualization over a variety of different types of datasets, and obtained excellent results when compared to the baseline methods.

The rest of this paper is organized as follows: Section 2 describes related work. Section 3 explains the essential concepts that help to understand this paper. Section 4 introduces each module of the dSAFNE framework in detail. Section 5 presents and analyses the experimental results. Section 6 summarizes the work in this paper.

## 2. Related Work

In this section, we briefly describe the related NRL approaches, which are classified into matrix-factorization-based models and shallow neural embedding models according to implementation characteristics.

The matrix-factorization-based models [17,18,19,20,21] first used different types of relational matrices [20] to preserve the network information, and then applied matrix factorization to obtain vertex representations. Belkin et al. [17] utilized Laplacian spectral decomposition to discover non-linear low-dimensional representations for the input network data. To learn community-oriented vertex representation, Tang et al. [18] performed a decomposition of the modularity matrix. Donnat et al. [19] embedded vertex neighborhood structure into a low-dimensional space and preserved structural role proximity by leveraging spectral graph wavelet diffusion patterns. Yang et al. [20] executed inductive matrix decomposition [22] on the vertex context matrix to preserve both network structure and vertex text attribute characteristics in the vertex representation vector. Zhang et al. [21] focused on the weakness of ignoring the homophily property in [20], and suggested a regularization term that simultaneously integrates homophily, structural context and vertex content to extract network representations. Although the matrix-factorization-based approach can effectively extract the global structural features of networks, these methods are challenging to be deployed to large networks since matrix factorization requires a great deal of computing and memory resources. In the meantime, the design of relational matrices will have a direct impact on the performance of matrix factorization, introducing an extra contingency.

The shallow neural embedding models [9,10,11,12,23,24,25,26] seek an embedding matrix, which can be considered as a vertex representation by a row or column vector. Typically, they first extract vertex sequences by performing random walks on the network to capture network structure. Based on the assumption that the comparable context-sharing vertices in random walk sequences should be closely represented in the low-dimension feature space, they then train a shallow neural language model on the sequences to optimize the embedding matrix, i.e., to maximize the log-likelihood of context vertices when given a target vertex. Perozzi et al. [9] performed truncated random walk on the network to generate vertex sequences, in which the co-occurrence frequencies of vertex-context pairs indicated their relevance. To further extract the vertex representation, they generalized the idea of a word embedding model (i.e., Skip-Gram [27]) over the vertex sequences. Cao et al. [23] followed the idea of [9] and extended the Skip-Gram model to capture *k*-step high-order vertex proximity, with the goal of making vertices that shared common *k*-step neighbors proximate in the embedding space. In contrast to the rigid strategy of truncated random that defines the neighborhood (context) for each vertex, Grover et al. [10] introduced a biased random walk to balance breadth-first search (BFS) and depth-first search (DFS) [28], and then applied Skip-Gram to learn the vertex feature from random walk sequences. To preserve the structural role proximity of vertices, Ribeiro et al. [11] first encoded vertex structural role proximity into a multi-layer graph, where the edge weights of each unique graph were defined by the structural role difference, and then executed [9] at each layer to learn vertex representations.

In order to incorporate vertex labels with network structure, Li et al. [24] proposed learning discriminative network representations by concurrently optimizing the objective of [9] and supporting vector classification. Pan et al. [25] adapted a paragraph vector model [29] to capture vertex textual content and label information, and then jointly optimized the objective of [9] to simultaneously encode network structure, vertex label and attribute information into a vertex representation. Sun et al. [12] exploited long short-term memory (LSTM) [30] to maintain the transfer possibilities among the vertices. To further improve the extraction of the network’s local structural features, they devised a Laplacian-supervised embedding space optimization. Rozemberczki et al. [26] proposed a Euler walk for executing a Euler tour in the diffusion subgraph centered on each vertex.

In comparison to matrix factorization-based models, shallow neural embedding models are more robust since they are unaffected by artificially constructed relation matrices. Moreover, since random walk can be easily applied to large-scale networks, the scalability of such approaches is considerably enhanced. Despite this, there still remain a series of shortcomings. These models do not consider the effect of a series of historical vertices on the sampling probability when performing random walks, leading to the loss of community structure. Limited by the feature extraction ability and receptive field of the shallow neural language model, only local structural features of the network can be extracted. In addition, it is necessary to design attribute fusion strategies specifically for a variety of downstream tasks and attribute types.

Motivated by the aforementioned investigations, we are committed to addressing the following issues of previous shallow neural embedding methods, including the loss of community structure resulting from the static-probability-distribution-based random walk, the inability to simultaneously learn multi-grained structural features, and the task-specific vertex attribute integration algorithm requirements.

## 3. Preliminaries

In this section, we summarize some essential concepts and define the research problem.

**Definition** **1** (Network)**.** *A network can be formally represented as a triplet G=(V,E,C), which consists of a vertex set $V=\{v_{1},v_{2},\cdots ,v_{n}\}$ of n vertices, an edge set $E={\left\{e_{ij}\right\}}_{i,j=1}^{n}$, and a vertex attributes set $C=\{c_{1},c_{2},\cdots ,c_{n}\}$ where $c_{i}$ denotes the attributes of vertex $v_{i}$. Each object $e_{ij}$ in E indicates that there exists an edge, which is attached with a weight $w_{ij}\in R$, from vertex $v_{i}$ to $v_{j}$. In an unweighted network, $w_{ij}=1$ if $e_{ij}\in E$ else $w_{ij}=0$.*

**Definition** **2** (Vertex Proximity)**.** *In real-world networks, many vertex proximities exist, including first-order, second-order, and higher-order proximities, etc. The first-order proximity function can be used to determine the direct connectedness of two vertices, which is often specified as the weight of the connecting edge. The second-order proximity of two vertices indicates the distance between their adjacent distributions [31]. Higher-order proximity between two vertices can be defined as the k-step probability of transitioning between them [32].*

**Definition** **3** (Structure and Vertex Attributes Fusion Network Embedding)**.** *The structure and vertex attributes fusion network embedding task attempts to learn a low-dimensional representation matrix $R\in R^{\left|V\right|\times d}$ for a given network G, where $d\ll \left|V\right|$. The row vector $R_{i,:}$ is treated as the representation of vertex $v_{i}$. $R_{i,:}$ should preserve both the structural information and vertex attribute information. In other words, the vertices that share common neighbors, or have similar attributes, should be represented closely in the low-dimension feature space.*

## 4. Approach

**Figure 2:**
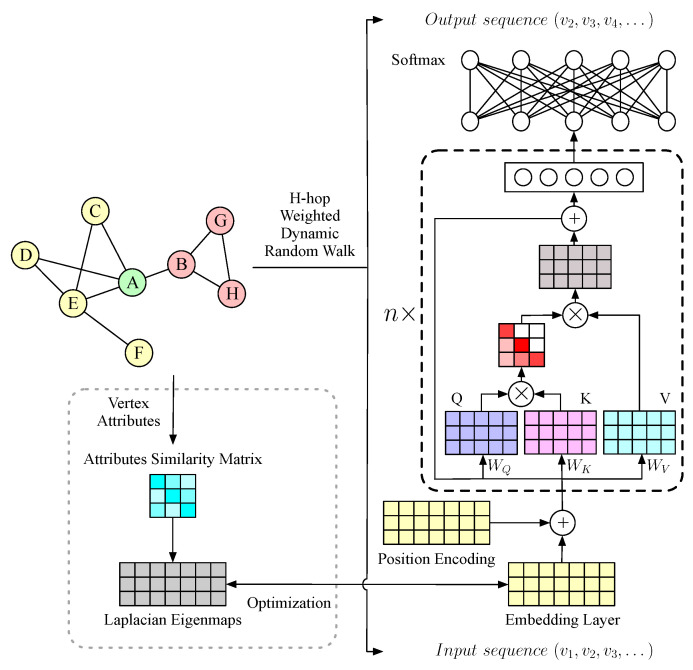
The overall framework of dSAFNE.

In this section, we present details of the proposed NRL approach dSAFNE. Figure 2 shows the overall framework, which consists of three modules: (a) an *h*-hop weighted dynamic random walk for sampling vertices sequences on the top left, (b) a novel masked self-attention-based deep neural prediction model for capturing mixed-grained structure on the right, (c) a vertex attributes-driven Laplacian space optimization for capturing vertex attribute features on the bottom left.

### 4.1. Random Walk

#### 4.1.1. Asymmetric Second-Order Proximity

**Figure 3:**
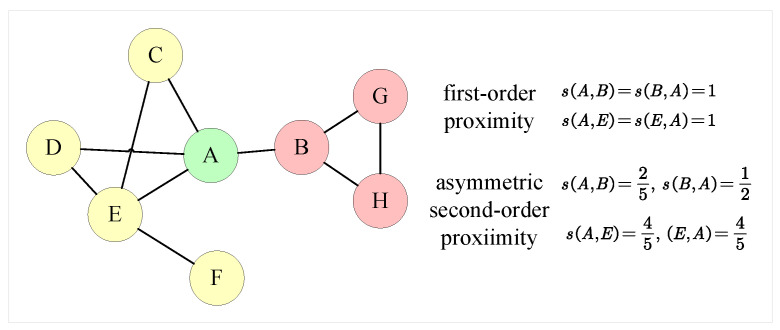
A toy example of a social network, where different colors indicate distinct communities. First-order proximity and asymmetric second-order proximity is quite different among vertices *A*, *E* and *B*.

Edges in networks always signify a degree of resemblance or connection between vertices. Traditional NRL approaches [9,33] always adopt first-order proximity to characterize the pairwise proximity. However, in an unweighted network, the symmetric first-order proximity between all directly connected vertices is equal to one, obliterating relationship information and social roles associated with vertices. Figure 3 depicts a toy example of a social network, with different colors denoting distinct communities. Vertices *A* have more similar social roles with *E* than *B*, since *A* and *E* have more neighbors, who are usually more critical users in social networks, and information is more likely to propagate between *A* and *E*. Additionally, the interaction between vertices is usually asymmetric. In the instance of vertices *A* and *B*, *A* has more neighbors than *B*, so *B* plays a minor role in *A*’s context and has less influence on *A*, while *A* plays a more important role in *B*’s context and has a greater influence on *B*.

To measure the connection between vertices appropriately while retaining social role information, we define an asymmetric second-order proximity based on the notion that the more 1-hop neighbors two vertices share, the more similar their social roles are. Formally, given a network G=(V,E,C), we define the 1-hop central neighborhood of vertex $v_{i}$ as $Nc_{v_{i}}^{1}=\left\{v_{i}\right\}\cup N_{v_{i}}^{1}$, where $N_{v_{i}}^{1}=\left\{v_{i1},v_{i2},\cdots ,v_{ik}\right\}$ and each $v_{ik}\in N_{v_{i}}^{1}$ is directly connected with $v_{i}$ via edge $e_{ik}\in E$. For each pair of vertices $<v_{i},v_{j}\in N_{v_{i}}^{1}>$, the asymmetric second-order proximity $s_{ij}$ is calculated as follows:

(1) $$s_{ij}=w_{ij}\cdot \frac{\left|Nc_{v_{i}}^{1}\cap Nc_{v_{j}}^{1}\right|}{\left|Nc_{v_{i}}^{1}\right|}$$

where $w_{ij}$ is the edge weight of $e_{ij}$. As seen in Figure 3, the asymmetric second-order similarity has the ability to reflect social role information and more correctly characterize vertex similarity.

#### 4.1.2. *h*-Hop Weighted Dynamic Random Walk

Formally, given a network G=(V,E,C) and a random-walked vertex sequence $X=\{x_{1},x_{2},\cdots ,x_{i-1}\}$, the generation of the *i*th vertex in the walk can be considered as a Markov process with the following distribution:

(2) $$P\left(x_{i}=v|x_{i-1}=u\right)=\left\{\begin{array}{cc} \frac{\pi_{uv}}{Z}\; & if\;e_{uv}\in E\\ 0\; & else \end{array}\right.$$

where $\frac{\pi_{uv}}{Z}$ is the normalized transition probability between vertex *u* and *v*, and *Z* is the normalizing constant. In some work [9,12], the static edge weight is treated as the transition probability. However, these methods ignore the correlation between walked history and the vertices to be sampled, leading to loss of essential information about the community structure. In order to steer the random walk process between a series of vertices with high similarity and capture the local community structure, we introduce an *h*-hop dynamic random walk based on the asymmetric second-order proximity.

**Figure 4:**
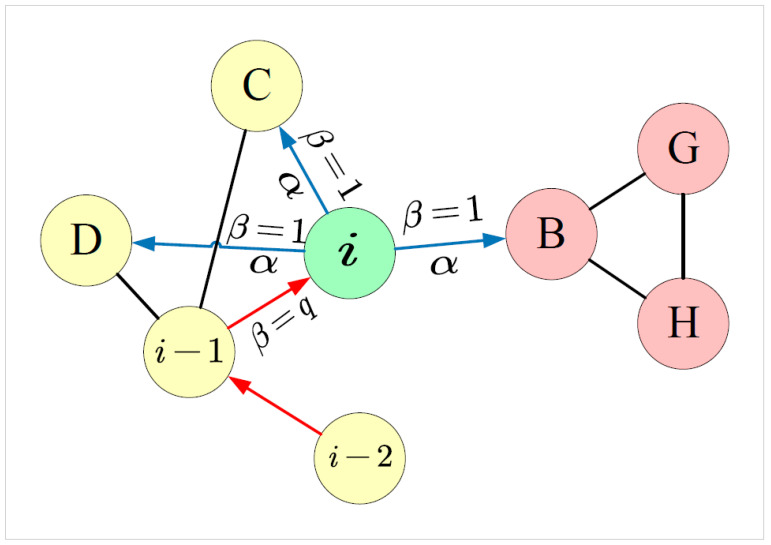
Illustration of the h-hop dynamic random walk procedure in dSAFNE. The walk transitioned from vertex $v_{i-2}$ to $v_{i}$ and is now evaluating its next step of vertex $v_{i}$. α and β indicate the decay parameter and retrospective parameter, respectively.

As shown in Figure 4, when calculating the transition probability $P(x_{i}=v|x_{i-1}=u)$, we consider the similarity among the *h* previously walked vertices $X_{h}=\left\{x_{i-h},x_{i-(h-1)},\cdots ,x_{i-1}\right\}$ and *v*. For each neighbor $v\in Nc_{x_{i-1}=u}^{1}$, the dynamic transition probability is calculated as follows:

(3) $$\begin{array}{c} P\left(x_{i}=v|x_{i-1}=u\right)=P\left(x_{i}=v|X_{h}\right)=\beta \frac{\sum_{j=1}^{h}\alpha^{i}s_{vx_{i-j}}}{Z} \end{array}$$

(4) $$\begin{array}{c} \beta =\left\{\begin{array}{cc} 1\; & v_{i}\neq u\\ q\; & v_{i}=u \end{array}\right. \end{array}$$

where $s_{vx_{i-j}}$ is the asymmetric second-order proximity between vertex $x_{i-j}$ and *v*, α∈(0,1) is the similarity decay parameter, which is used to adjust the degree to which past vertices have an effect on the transition probability. The greater alpha is, the more likely it is to walk among vertices that are similar to those in the historical vertex sequence, hence preserving more community structure information in the vertex sequence. β∈(0,1] is the retrospective parameter, which can control whether the walk goes backwards or not. When q→0, the walk approximates DFS behavior; when q→1, the walk approximates BFS behavior. The pseudocode for *h*-hop dynamic random walk is given in Algorithm 1.
**Algorithm 1:** The *h*-hop dynamic random walk procedure.
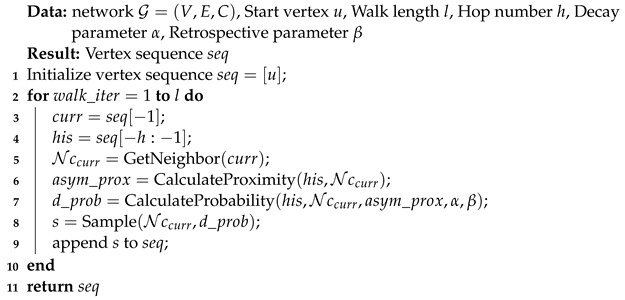


### 4.2. Deep Latent Structure Feature Extraction

The *h*-hop dynamic random walk process can be thought of as a message propagation process, where messages are more likely to spread inside the community, i.e., among vertices with high similarities. The random walk sequence can be seen as a time series of the message propagation path, i.e., a projection of the structure of the network. Following the assumption of [9], we assume that, in the random walk sequences, the vertices sharing more common historical sequences should be presented closely in the embedding space, i.e., they should have similar structural features. Therefore, we can extract the multi-grained structural features by modeling the co-occurrence relationship between the current vertex and the short-range/long-range historical vertices. With such a hypothesis, we anticipate maximizing the likelihood of the current vertex when given historical walked vertices. Given a random walk sequence $X=\{x_{1},x_{2},\cdots ,x_{l}\}$, the likelihood function is defined as follows:

(5) $$D\left(X\right)=\sum_{i=2}^{l}P\left(x_{i}|x_{1},\cdots ,x_{i-1}\right),W)$$

where *W* means the trainable model parameters.

Recently, Transformer [16] and its variations [34,35] have demonstrated promising performance on a variety of sequence prediction tasks, due to the powerful ability of the self-attention mechanism to model short- and long-range dependencies, which can be utilized to learn the local and global structure feature of network simultaneously. Thus, inspired by Transformer, we present a novel masked self-attention-based prediction model, which consists of an input layer, *n* encoder layers and an output layer. The detailed information is shown in the right of Figure 2. In contrast to the traditional Transformer, we omit the decoder and, instead, feed the encoder’s output directly into a softmax layer, and output the prediction probability of the target vertex. By converting the encoder’s self-attention layer to a one-way masked self-attention, we expect the prediction model to automatically learn the correlation between the target vertex and the historical vertices. When given a network G, we initially represent vertices with an embedding matrix $R\in R^{|V|\times d}$, where each row $R_{i,:}$ indicates the representation of vertex $v_{i}$. To enable the model to utilize the temporal information included in the vertices’ sequences, we follow the original design of the Transformer encoder to combine the positional encoding with the vertex embedding as the model’s input. The position encoding is used to identify the position of a vertex in the sequence. The positional encoding and the input of our model are calculated as follows:

(6) $$\begin{array}{c} PE\left(pos,2i\right)=sin\left(\frac{pos}{10000^{2i/d_{model}}}\right) \end{array}$$

(7) $$\begin{array}{c} PE\left(pos,2i+1\right)=cos\left(\frac{pos}{10000^{2i/d_{model}}}\right) \end{array}$$

(8) $$\begin{array}{c} h_{0}=T_{S}R+PE \end{array}$$

where pos is the index of a vertex in the walk sequence, *i* indicates a dimension of the representation vector, $T_{S}\in R^{\left|V\right|\times \left|V\right|}$ is the one-hot tokenized matrix of vertices, $T_{i,:}$ means the one-hot encoding for vertex $v_{i}$, and $h_{0}$ is then fed into the encoder layer of the prediction model.

To improve the model’s capacity for deep latent feature extraction, we stack $n_{layer}$ encoder layers and leverage the output of the final encoder layer as the retrieved vertex temporal feature to predict the target vertex. The *l*-th encoder layer is formulated as follows:

(9) $$\begin{array}{c} Q_{l}=W_{l}^{q}h_{l-1} \end{array}$$

(10) $$\begin{array}{c} K_{l}=W_{l}^{k}h_{l-1} \end{array}$$

(11) $$\begin{array}{c} V_{l}=W_{l}^{v}h_{l-1} \end{array}$$

(12) $$\begin{array}{c} attn_{l}=I^{\prime }⊙softmax\left(\frac{Q_{l}K_{l}^{T}}{\sqrt{d_{k_{l}}}}\right) \end{array}$$

(13) $$\begin{array}{c} h_{l}^{\prime }=attn_{l}V_{l} \end{array}$$

(14) $$\begin{array}{c} h_{l}=h_{l-1}+h_{l}^{\prime } \end{array}$$

(15) $$\begin{array}{c} s.t.\;\forall l\in [1,n_{layer}] \end{array}$$

where $I^{\prime }$ is a lower triangular unit matrix, $W_{l}^{q}$, $W_{l}^{k}$ and $W_{l}^{v}$ are the trainable projection weights of *l*-th layer, $d_{k_{l}}$ is a normalization parameter, and $h_{l}$ indicates the hidden state of *l*-th layer encoder layer and is considered as the input of next encoder layer. The lower triangular matrix $attn_{l}\in R^{\left|X\right|\times \left|X\right|}$ is the values of the so-called attention of *l*-th layer, and $attn_{l(i,j)}$ denotes the influence of the *j*-th vertex in extracting the structural feature of the *i*-th vertex. The output of the $n_{layer}$-th encoder layer is treated as the deep latent feature to be fed into the output layer and predict the target vertex. The softmax output layer is defined as:

(16) $$\begin{array}{c} \hat{y}=softmax\left(h_{n_{layer}}\right) \end{array}$$

where $\hat{y}$ is the predicted probability distribution of the target vertex. We leverage the cross entropy [36] loss function $L_{pred}$ to measure the degree of asynchronism between $\hat{y}$ and the true probability distribution *y*, which is calculated as follows:

(17) $$L_{pred}=-\sum_{i}y_{i}*log\left(\hat{y_{i}}\right)$$

It is worth emphasizing that this is only a fake task [13] due to the goal of network representation learning. We just optimize the vertex representations through the prediction procedure, while the optimized embedding matrix *R* that encodes the deep latent structure feature of vertices is what is needed.

### 4.3. Vertex Attributes-Driven Laplacian Space Optimization

Although we are able to learn the local and global structural properties of the vertices using the prediction model, there may be topologically distant vertices with comparable attributes in the real network. Since vertex attributes typically contain extensive semantic information and can directly reflect the similarity of vertices at the attribute level, we should ensure that vertices with comparable attributes, but which are topologically distant, also remain adjacent in the embedding space.

To encode additional attribute features into the vertex representation while ensuring the versatility of our model, inspired by [12,37], we propose an attributes-driven Laplacian space optimization (Attr-LapSO) to extract attribute features. For the attributes of different types, we first extract their latent semantic features via the existing state-of-the-art learning models, such as ResNet [14] for images, BERT [15] for text, and multilayer perceptron (MLP) [38] for numerical attributes. We then calculate the attributes similarity matrix $F\in R^{\left|V\right|\times \left|V\right|}$ based on the extracted attribute feature under a specific criterion, e.g., cosine similarity or Euclidean distance, where each element $F_{i,j}$ of *F* indicates the attribute similarity of vertices $v_{i}$ and $v_{j}$. After that, we select the top *k* similar and dissimilar vertices as set $optim_{v_{i}}$ for each vertex $v_{i}$ in the network. Then, we use this similarity matrix to optimize the embedding matrix *R*. The loss function $L_{lap}$ of Attr-LapSO is defined as follows.

(18) $$\begin{array}{cc} L_{lap} & =\sum_{i=0}^{\left|V\right|}\sum_{j=1}^{2k}{\left(r_{i}-r_{j}\right)}^{2}F_{i,j} \end{array}$$

(19) $$\begin{array}{cc} \; & =2*Tr(R^{T}LR) \end{array}$$

where $v_{j}\in optim_{v_{i}}$, $r_{i}\in R$ is the representation of vertex $v_{i}$, L=D−F is a Laplacian eigenmap, $D\in R^{\left|V\right|}$ is a diagonal matrix, $D_{ii}=\sum_{j}F_{ij}$.

### 4.4. Model Training

The pseudocode of dSAFNE is presented in Algorithm 2. To learn model parameters, we take advantage of a backward propagation and mini-batch Adam optimization scheme [39] to minimize the loss functions $L_{pred}$ and $L_{lap}$, and to update the model parameters. As demonstrated in lines 11→16 of Algorithm 2, the two loss functions are alternatively and iteratively optimized for primarily two reasons. On the one hand, combining the two loss functions will make it difficult to update the parameters of the prediction model. On the other hand, by sharing the vertices representation vectors across the two stages, one stage’s training helps accelerate the other’s convergence process.
**Algorithm 2:** The dynamic structure and vertex attributes fusion network embedding framework.
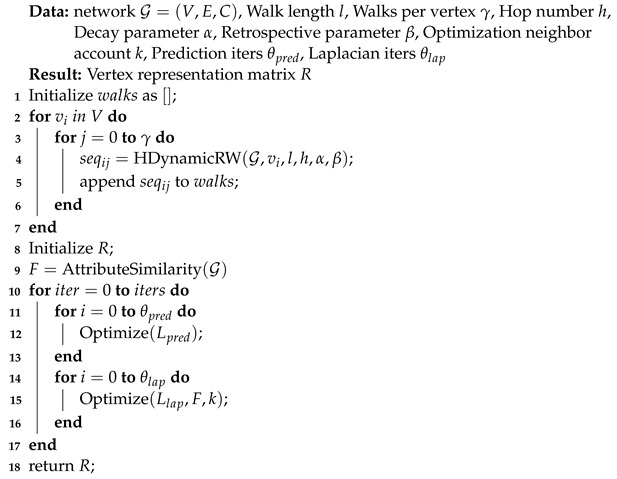


## 5. Evaluation

### 5.1. Experimental Setup and Datasets

Our experiments were conducted on a workstation outfitted with an Intel Xeon Gold 6132 processor running at 2.60 GHz, an NVIDIA Geforce GTX 1080Ti graphics card, and 192 GB of RAM. The Python 3.8.5 and Pytorch 1.11.0 were utilized to develop the dSAFNE framework. The code is available at https://github.com/SXiangHu/dSAFNE, accessed on 24 July 2022.

To examine the proposed dSAFNE framework thoroughly, we undertook experiments on five benchmarking datasets of varying sizes. Table 1 presents detailed statistics for the datasets.

- Facebook [40] is a network of verified Facebook sites. The vertices indicate official Facebook pages, which are classified into one of four categories. Each vertex is attached with a series of keywords extracted from the site description as its attribute. The vertex attribute is represented as a binarization vector by the bag-of-words model. The edges represent mutual likes.
- BlogCatalog [18] is a network of bloggers, with vertices representing bloggers and edges representing the relationships of friendship between them. Each vertex is associated with at least one label.
- 3-Cora, CiteSeer, and PubMed [3] are networks in which vertices represent scientific publications and edges denote citation relationships. According to their research topic, vertices are classified into various categories. The vertex attribute is the abstract of a publication.

### 5.2. Baseline Methods

The baseline methods are listed as follows:

- DeepWalk [9]: DeepWalk pioneers the use of language modeling with unsupervised feature learning to learn latent representations of vertices, achieving SOTA results at that time.
- TADW [20]: Text-attributed deep walk (TADW) leverages matrix factorization to incorporate vertex rich text attributes into the embedding process.
- Node2vec [10]: Node2vec defines a flexible concept of a vertex’s network neighborhood and implements a biased random walk technique that explores diverse neighborhoods to learn richer representations.
- Struc2vec [11]: Struc2vec creates a multi-layer graph to encode structural similarity and produce structural context for vertices, and then learns the latent representations for the vertices’ structural identities.
- DNE [12]: Deep network embedding (DNE) proposes an LSTM-based deep embedding framework that preserves transfer possibilities between network vertices and uses Laplacian supervised space optimization to capture the local structure.
- Attributes: The features of vertex attributes are treated directly as the vertex representations.

### 5.3. Parameter Setting

The dimensions of the extracted vertex representations were set to 128 for all datasets. We adhered to the parameter settings indicated in the appropriate literatures for baseline approaches. For the proposed dSAFNE framework, we set the walk length and walks per vertex to 100, and we set the number of encoder layers $n_{layer}=8$. At the stage of model training, we raised the learning rate from zero to $10^{-3}$ linearly over the first 5000 steps, and then annealed it to $10^{-4}$ by an exponential scheduler. For all datasets excluding BlogCatalog, we adopted denoising autoencoder (DAE) [41] to extract the vertices’ attribute features and calculate the cosine similarities between each pair of vertices.

**Figure 5:**
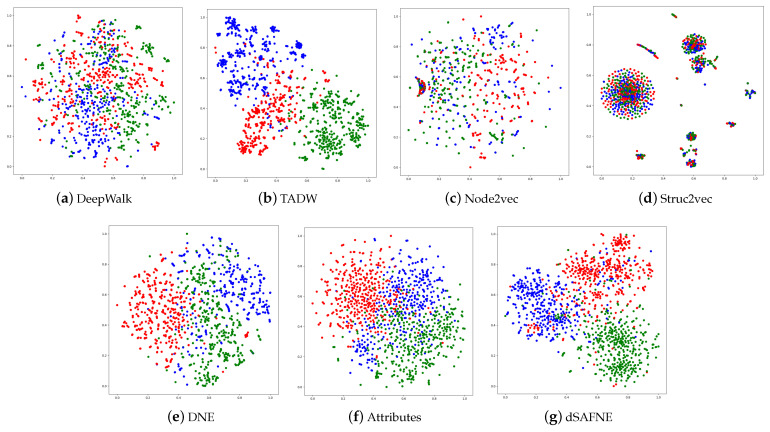
Visualization of 3-Cora. Every point represents a scientific publication. Different colors correspond to distinct categories. Red denotes neural network, blue represents rule learning, and green means reinforcement learning.

We intended to disclose the network structure in this task by intuitively viewing the acquired vertex representations. If the vertex representations are sufficiently representational, vertices of the same class should be close to each other, while vertices of different classes should be far from each other in the embedding space. We trained the proposed dSAFNE model and baseline approaches on the 3-Cora dataset to learn vertex representations. The vertices in 3-Cora were classed as neural network, rule learning, or reinforcement learning. Then we used t-SNE [42] to project the vertex representation learned through various approaches onto a two-dimensional space and visualize them, as depicted in Figure 5.

### 5.4. Experimental Results and Analysis

#### 5.4.1. Visualization

The visualizations of DeepWalk, Node2vec and Struc2vec were meaningless since points belonging to the same category are not clustered together, while DNE was capable of clustering the majority of points with the same label, but the borders were not obvious enough. TADW achieved the best visualization results among the baseline approaches because of the incorporation of vertex text attribute information in the process of representation learning. Even so, dSAFNE performed significantly better than the baseline approaches. Our method was capable of clustering not only points belonging to the same category but could also clearly separate clusters. This experiment demonstrated that dSAFNE is capable of learning representations that are more resilient and informative.

#### 5.4.2. Node Classification

In this part, we performed multi-label classification on the BlogCatalog dataset, followed by multi-class classification on the Facebook, CiteSeer, and PubMed datasets. The representations gained through different approaches are referred to as the vertices feature vectors. For each dataset, a random sample of the labeled vertices was used as training data to train an MLP classifier, while the remaining vertices were used as test data. We measured the performance of classification results via the widely adopted accuracy, micro-F1 (miF1) and macro-F1 (maF1) scores, which are defined as follows:

(20) $$\begin{array}{c} accuracy=\frac{\sum_{i=1}^{r}TP_{i}}{\left|D\right|} \end{array}$$

(21) $$\begin{array}{c} miP=\frac{\sum_{i=1}^{r}TP_{i}}{\sum_{i=1}^{r}\left(TP_{i}+FP_{i}\right)} \end{array}$$

(22) $$\begin{array}{c} miR=\frac{\sum_{i=1}^{r}TP_{i}}{\sum_{i=1}^{r}\left(TP_{i}+FN_{i}\right)} \end{array}$$

(23) $$\begin{array}{c} miF1=2\frac{miP\times miR}{miP+miR} \end{array}$$

(24) $$\begin{array}{c} P_{i}=\frac{TP_{i}}{TP_{i}+FP_{i}} \end{array}$$

(25) $$\begin{array}{c} R_{i}=\frac{TP_{i}}{TP_{i}+FN_{i}} \end{array}$$

(26) $$\begin{array}{c} maF1=\frac{1}{r}\sum_{i=1}^{r}F1_{i}=\frac{2}{r}\sum_{i=1}^{r}\frac{P_{i}\times R_{i}}{P_{i}+R_{i}} \end{array}$$

where *r* means the number of categories, |D| indicates the number of samples in dataset D. $TP_{i}$, $FP_{i}$ and $FN_{i}$ denote the number of true positive predictions, the number of false positive predictions and the number of false negative predictions, respectively, for category *i*. Accuracy and micro-F1 scores indicate the overall performance of various approaches, while macro-F1 emphasizes the performance in rare categories. We repeated each task ten times and analyzed the experimental results using an ANOVA test.

On the BlogCatalog dataset, we exclusively focused on learning network structural features to demonstrate the *h*-hop dynamic random walk and masked self-attention based prediction model’s efficacy at capturing network structure information. We excluded TADW because it uses DeepWalk to maintain the network structure. From Table 2, one can see that dSAFNE improved the best baseline DNE by 7.67% (Micro-F1) and 4.26% (Macro-F1), respectively, and achieved a 20.43% (Micro-F1) and 18.47% (Macro-F1) increase. From the experimental results, we can be sure that the *h*-hop dynamic random walk and self-attention mechanism can incorporate additional contextual information, which enables the model to retain more information about the network’s global and local structure.

Table 3 shows the results of multi-class classification. As can be seen, dSAFNE routinely outperformed the other baseline methods. dSAFNE improved accuracy by 4.42% on Facebook, 4.75% on CiteSeer, and 4.83% on PubMed with 30% labeled vertices when compared to the best baseline TADW. The classification results suggest that dSAFNE is effective at taking into account the network structure and vertex attribute information in a comprehensive manner, significantly improving the quality of the learned representation vectors.

#### 5.4.3. Parameter Sensitivity

This section investigates the method’s sensitivity to various hyperparameter values. The proposed dSAFNE mainly takes seven hyperparameters into account: the dimension of the learned representation vector *d*, walks per vertex γ, walk length *l*, decay parameter α, retrospective parameter β, number of prediction model training steps per iteration $\theta_{pred}$ and Laplacian space optimize steps per iteration $\theta_{lap}$. To determine the optimal values for various hyperparameters, we assessed the multi-class classification performance with varying parameter settings, changing the value of one parameter at a time. We randomly selected 30% of the CiteSeer dataset’s vertices as the training set and the remaining 70% as the test set.

**Figure 6:**
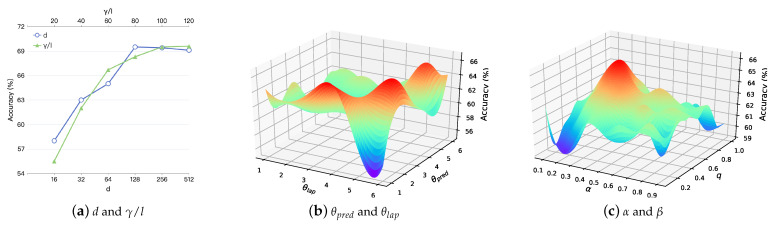
Accuracy scores for different parameters on CiteSeer.

Figure 6a illustrates the influence of various *d* and γ/l values on the performance of classification. For convenience, γ is set equal to *l*. As *d* increases, the accuracy increases initially and then maintains relative stability because the vector can keep more information as the dimension increases. When d=128, the classification accuracy is at its maximum. The figure demonstrates that as γ and *l* grow, the classification accuracy increases as well, because γ and *l* correlate to the corpus’s richness. The corpus is more abundant when γ and *l* have bigger values. Since the self-attention mechanism is capable of resolving the problem of long-distance dependency, increasing the walk length has no adverse effect and also better captures the network’s global structure.

Figure 6b shows the accuracy scores for different values of the number of prediction model training steps per iteration $\theta_{pred}$ and Laplacian space optimize steps per iteration $\theta_{lap}$. The accuracy score is maximized when $\theta_{pred}=1,\theta_{lap}=3$, the two training procedures are balanced, and the model converges effectively. As $\theta_{pred}$ increases, the model loses the information about vertex attributes, and as $\theta_{lap}$ increases, the model tends to converge the absolute values of each dimension of the representation to 0.

Figure 6c depicts the accuracy scores for different values of the decay parameter α and retrospective parameter β. When α=0.5,β=0.2, the accuracy score has a maximum value. With increasing α, the context vertices exert a bigger effect on the random walk process, and the sampled vertex sequences contain more information about local community structure. However, if α is set to a value that is too large, the random walk process will lack randomization, preventing it from capturing different types of neighbor vertices, and, therefore, coarse-grained structural features of the network will be lost. β can be used to adjust the bias of the random walk process for depth-first or breadth-first search. The greater the β, the more breadth-first search is favored, while the lower the β, the more depth-first search is favored. When β=0.2, the walk procedure can better balance DFS and BFS, thus capturing different types of structural features.

Considering both effectiveness and efficiency, Table 4 summarizes the optimal values of key hyperparameters for our experimental scenarios.

## 6. Summary and Discussion

This paper proposes a general NRL framework dSAFNE, which focuses on the shortcomings of previous NRL methods, including the excessive randomness of the random walk process based on symmetric node similarity and static probability distribution, the inability to simultaneously learn multi-grained structural features, and inefficient vertex attribute utilization. The proposed dSAFNE framework mainly consists of three modules: an *h*-hop weighted dynamic random walk, a masked self-attention-based prediction model and a vertex attributes-driven Laplacian space optimization. Based on the well-designed asymmetric second-order similarity, which reflects vertex similarity while maintaining social role information, the proposed *h*-hop weighted dynamic random walk can capture the community structure information effectively. Due to the wide receptive field of the self-attentive mechanism, the prediction model can simultaneously learn the co-occurrence relationship between the target vertex and the short-range/long-range contextual vertices, leading to the extraction of multi-grained structural features. In addition, the proposed vertex-attributes-driven Laplacian space optimization paradigm makes it simple to integrate different types of vertex attributes into the vertex representation, hence ensuring the future generalizability of the model. Furthermore, extensive experiments were conducted on five benchmarking datasets. The experimental results demonstrated that dSAFNE is capable of learning more representational vertex features than the state-of-the-art competing approaches.

While these experimental results are encouraging, some limitations of the proposed dSAFNE framework remain. On the one hand, similar to the regular Transformer, the self-attention-based prediction model suffers from the quadratic complexity with the length of random walk sequence, which restricts the possibility to capture the global structure of large-scale networks by extending the length of random walk sequences, particularly in scenarios where computational resources are constrained. Therefore, the development of a more efficient prediction model and network sampling strategies are necessary. On the other hand, the present version of dSAFNE is only applicable to homogeneous networks and does not account for the heterogeneity of vertices and edges. It is crucial to extend dSAFNE to heterogeneous networks in order to improve the generalizability. Finally, in addition to node visualization and classification, NRL has a vast array of downstream tasks, such as link prediction, temporal prediction and recommender system, etc. The efficiency of the proposed dSAFNE framework with respect to these downstream tasks needs to be further explored. We leave this for future investigations.

## Figures and Tables

**Table 1:** Statistics of different datasets.

Dataset	Vertices	Edges	Categories
Facebook	22,470	171,002	4
BlogCatalog	10,312	333,983	39
3-Cora	1195	3429	3
CiteSeer	3311	4732	6
PubMed	19,717	44,338	3

**Table 2:** Micro-F1/Macro-F1 scores for multi-label classification on BlogCatalog; * indicates that our model significantly outperformed the best baseline based on ANOVA test (p<0.05).

	Algorithm	%Labeled Vertices		
		10%	20%	30%
Micro-F1	DeepWalk	33.17 ± 1.21	35.84 ± 2.31	37.28 ± 0.23
	Node2vec	31.25 ± 2.07	32.27 ± 1.80	32.24 ± 1.60
	Struc2vec	11.16 ± 2.29	11.20 ± 0.63	12.86 ± 0.20
	DNE	34.13 ± 2.18	37.25 ± 2.23	37.56 ± 2.32
	dSAFNE	39.54 * ± 2.10	44.25 * ± 1.15	45.23 * ± 2.36
	Gains	15.85%	18.79%	20.43%
Macro-F1	DeepWalk	17.40 ± 0.45	20.39 ± 2.63	21.96 ± 2.38
	Node2vec	11.54 ± 1.33	14.33 ± 0.58	16.31 ± 1.05
	Struc2vec	5.24 ± 2.73	5.57 ± 0.05	4.83 ± 2.34
	DNE	17.38 ± 0.58	21.41 ± 2.48	23.06 ± 0.92
	dSAFNE	21.04 * ± 2.00	25.31 * ± 0.15	27.32 * ± 2.62
	Gains	21.05%	18.21%	18.47%

**Table 3:** Accuracy scores for multi-class classifications on Facebook, CiteSeer and PubMed; * indicates that our model achieved significant improvements.

Algorithm	Facebook			CiteSeer			PubMed		
**%Labeled Vertices**	**10%**	**20%**	**30%**	**10%**	**20%**	**30%**	**10%**	**20%**	**30%**
DeepWalk	72.09 ± 1.22	74.83 ± 2.38	76.14 ± 1.13	50.94 ± 1.25	51.54 ± 1.78	53.28 ± 1.47	69.94 ± 1.27	71.37 ± 2.20	72.51 ± 1.41
TADW	79.48 ± 1.56	80.50 ± 1.25	83.08 ± 2.01	60.89 ± 1.83	62.32 ± 1.36	64.78 ± 0.62	80.63 ± 2.39	81.76 ± 0.61	84.32 ± 1.03
Node2vec	53.44 ± 0.98	54.81 ± 0.22	55.81 ± 1.00	30.41 ± 1.31	32.12 ± 1.11	32.75 ± 0.49	39.04 ± 2.20	39.37 ± 0.25	39.83 ± 2.27
Struc2vec	35.58 ± 1.39	35.13 ± 1.05	35.82 ± 1.95	25.01 ± 2.33	26.76 ± 1.04	27.75 ± 0.19	47.08 ± 1.53	48.15 ± 0.56	49.32 ± 2.16
DNE	68.06 ± 0.66	70.62 ± 0.47	73.06 ± 0.02	50.56 ± 1.51	52.97 ± 1.39	54.04 ± 1.36	73.11 ± 1.40	73.04 ± 0.29	74.72 ± 0.51
Attributes	74.27 ± 0.14	76.62 ± 2.23	78.41 ± 0.58	57.31 ± 1.84	59.20 ± 0.92	61.09 ± 1.30	75.45 ± 1.65	77.52 ± 1.10	78.79 ± 0.84
dSAFNE	84.23 * ± 2.10	85.88 * ± 0.38	87.50 * ± 0.90	65.38 * ± 1.47	68.13 * ± 1.36	69.53 * ± 1.41	85.27 * ± 1.74	87.04 * ± 2.07	89.15 * ± 1.22
Gains	5.97%	6.68%	5.35%	7.37%	9.32%	7.33%	5.75%	6.45%	5.73%

**Table 4:** Optimal values of various hyperparameters.

Hyperparameter	Description	Value
*d*	Dimension of the learned representation vector	128
γ	Walks per vertex	100
*l*	Walk length	100
$\theta_{pred}$	Number of prediction model training steps per iteration	1
$\theta_{lap}$	Number of Laplacian space optimize steps per iteration	3
α	Decay parameter	0.5
β	Retrospective parameter	0.2

## Data Availability

Not applicable.

## Abbreviations

The following abbreviations are used in this manuscript:

NRL	Network representation learning
dSAFNE	Dynamic structure and vertex attributes fusion network embedding
Attr-LapSO	Attributes-driven Laplacian space optimization
LSTM	Long short-term memory
BERT	Bidirectional encoder representations from transformer
MLP	Multilayer perceptron
TADW	Text-attributed deep walk
DNE	Deep network embedding
SOTA	State-of-the-art
